# Multi-omics modality completion and knowledge distillation for drug response prediction in cervical cancer

**DOI:** 10.3389/fonc.2025.1622600

**Published:** 2025-08-27

**Authors:** DongZi Li, Bowei Yan, Kai Liao, Jian Huang, Jing Zhang, YiChen Chen, Jue Zhu, Shuang Zhi, Liping Chen

**Affiliations:** ^1^ Department of Gynecology and Obstetrics, The Affiliated Women and Children’s Hospital of Ningbo University, Ningbo, China; ^2^ Institutes of Biomedical Sciences, Fudan University, Shanghai, China; ^3^ The Central Laboratory of Birth Defects Prevention and Control, The Affiliated Women and Children’s Hospital of Ningbo University, Ningbo, China; ^4^ Ningbo Key Laboratory for the Prevention and Treatment of Embryogenic Diseases, The Affiliated Women and Children’s Hospital of Ningbo University, Ningbo, China; ^5^ Ningbo Key Laboratory of Genomic Medicine and Birth Defects Prevention, The Affiliated Women and Children’s Hospital of Ningbo University, Ningbo, China; ^6^ College of Computer Science, Chongqing University, Chongqing, China; ^7^ Basic Research Laboratory, The Affiliated Women and Children’s Hospital of Ningbo University, Ningbo, China

**Keywords:** multi-omics, cervical cancer, drug response prediction, knowledge distillation, modality completion

## Abstract

In clinical practice, the development of personalized treatment strategies for cervical cancer is hindered by the limited accuracy of drug response prediction, partly due to missing modalities in multi-omics data. We present MKDR, a deep learning framework that integrates variational autoencoder-based modality completion with knowledge distillation to transfer information from complete omics data to incomplete samples. MKDR-Student achieves state-of-the-art performance On cervical cancer cell lines, with an MSE of 0.0034 (34% lower than Xgboost), R² of 0.8126, and MAE of 0.0431, while maintaining high Spearman (0.8647) and Pearson (0.9033) correlations. Data ablation experiments highlight the contributions of knowledge distillation and modality completion: removing the teacher increases MSE by 23%, and VAE reduces error by 15% with 40% missingness. Interpretability analysis shows balanced feature contributions from gene expression (38%), copy number variation (30%), and mutation data (32%), indicating effective multi-omics learning and integration by the student model. Under limited-input conditions, MKDR’s accuracy drops less than 5%, supporting its robustness and potential for clinical application.

## Introduction

Cervical cancer remains a major malignancy among women worldwide, with approximately 604,000 new cases and 341,000 deaths reported in 2020 (1). Despite progress in HPV vaccination and early screening, significant variability in treatment response persists due to the high heterogeneity of molecular subtypes and the tumor microenvironment. Precision medicine aims to tailor therapies based on individual molecular profiles, with drug response prediction (DRP) playing a key role in estimating treatment efficacy before administration (2). Recently, various deep learning approaches have been proposed to improve prediction accuracy and safety by integrating multi-omics data and drug structures (3).

Furthermore, integrating multi-omics data—such as gene expression, DNA methylation, and mutation profiles—can improve drug response prediction by providing a more comprehensive view of tumor biology (4, 5). Models such as GADRP (6), DeepCDR (7), and MOLI (8) have shown strong performance through multi-omics integration. However, these models generally assume complete data across all modalities, limiting their robustness in real-world scenarios. For example, GADRP uses graph convolutional networks and autoencoders to integrate omics data with drug structures for drug response prediction. While effective on complete datasets, its performance drops sharply when methylation data are missing (6). Similarly, DeepCDR employs a hybrid GCN framework and achieves a PCC of 0.923 on the GDSC dataset, but it heavily relies on methylation features and lacks robustness under missing-data conditions (7). As a late-fusion model, MOLI requires complete input across all omics modalities; when data are missing, imputation is used, which introduces accumulated errors and increases RMSE by up to 15% (8). BANDRP integrates gene expression, mutation, methylation, and pathway features with drug fingerprints using bilinear attention. Despite strong predictive performance (PCC = 0.9382), it shows limited robustness when key omics modalities are missing (9). However, in real-world clinical settings—especially when using patient-derived samples—the acquisition of complete multi-omics data is often limited by high cost, technical barriers, and biological complexity (4, 10). As a result, many samples exhibit missing modalities or insufficient sequencing depth, particularly in valuable human-derived tissues. For example, although TCGA has profiled over 11,000 cancer cases, only a small subset includes complete multi-omics layers such as genomics, transcriptomics, methylation, and proteomics (11). This problem is more severe in female-specific cancers like cervical cancer, where full multi-omics profiles are especially rare (12). Moreover, variability in sequencing depth further complicates integration. In proteomics, for instance, up to 50% of peptide measurements may be missing due to inconsistent detection during mass spectrometry (13–16). These challenges significantly hinder the development of robust drug response models, as missing modalities often contain essential biological information.

Current approaches for handling modality missingness, such as data imputation and multimodal fusion networks, have achieved success in image recognition and natural language processing. However, they still face significant challenges in drug response prediction (17, 18). The fundamental reason lies in the fact that the correlations between clinical omics modalities are heavily influenced by biological heterogeneity (18). High-dimensional omics data are inherently complex and costly to acquire, and their missingness is often non-random (missing not at random, MNAR). For example, mutation status can be decisive for targeted therapy responses, while methylation patterns are closely associated with drug resistance mechanisms (19, 20). Modality missingness weakens signal recognition and increases bias, compromising the reliability of personalized treatment (8, 21). However, most drug response models assume complete omics data, overlooking real-world missingness and limiting clinical applicability. Knowledge distillation (22), originally developed for model compression, has recently been applied to multimodal learning to enhance generalization under missing modality conditions. For instance, KL4MTL (Ahn et al., 2019) (23) introduced a KD framework in multi-task learning to enforce consistent representations across tasks. In parallel, generative approaches such as MIDAS (24) employ variational autoencoders (VAEs) to impute missing omics modalities in single-cell data, demonstrating the feasibility of cross-modality reconstruction. More recently. MCKD (25) integrates KD with cross-modal meta-learning by adaptively weighting the importance of each modality, achieving robust prediction under partial modality missingness. However, these methods still fall short in addressing the unique challenges of drug response prediction in clinically realistic scenarios, where data are scarce and biologically heterogeneous.

To address these challenges, we propose MKDR, a modular deep learning framework that integrates a VAE-based modality completer, a knowledge distillation module, and a cross-modal attention mechanism. This architecture enables the student model to make accurate predictions under incomplete omics conditions by learning from a teacher model trained on complete data. Notably, MKDR achieves strong performance even when only a single omics modality is available, with MKDR-Student reaching a PCC of 0.9033 in cervical cancer drug response prediction. These results demonstrate the framework’s potential as a generalizable and deployable solution for real-world precision oncology scenarios.

## Materials and methods

### Datasets

This study constructs a drug response prediction model based on high-quality public datasets by integrating multi-omics profiles from the Cancer Cell Line Encyclopedia (CCLE) and drug response data from the PRISM Repurposing dataset. CCLE provides comprehensive omics data, including copy number variation (CNV), mutation (MU), and gene expression (GE), with 761, 7806, and 16,384 features respectively, to characterize the molecular states of cancer cell lines (26).The PRISM dataset contains IC_50_ measurements for 1,448 compounds tested across 578 human cancer cell lines, representing one of the largest high-throughput drug sensitivity screening platforms available to date (6, 27). To assess generalizability beyond the PRISM training cohort, we additionally conducted external validation experiments using the GDSC (28) SISO cervical cancer cell line and the TCGA-CESC (29) patient cohort.

This study focuses on 15 cervical cancer cell lines, from which a total of 6,935 valid drug–cell line response pairs were extracted from the PRISM dataset. To eliminate scale differences and enhance model stability, IC_50_ values were log-transformed, and outliers were removed using the interquartile range (IQR) method, which is widely adopted in related studies (6). The molecular structures of drugs were represented using canonical SMILES strings and standardized with the RDKit toolkit. The SMILES sequences were then tokenized to train a sequence-based molecular representation model.

## Methods

### Overview of the MKDR framework

**Figure 1:**
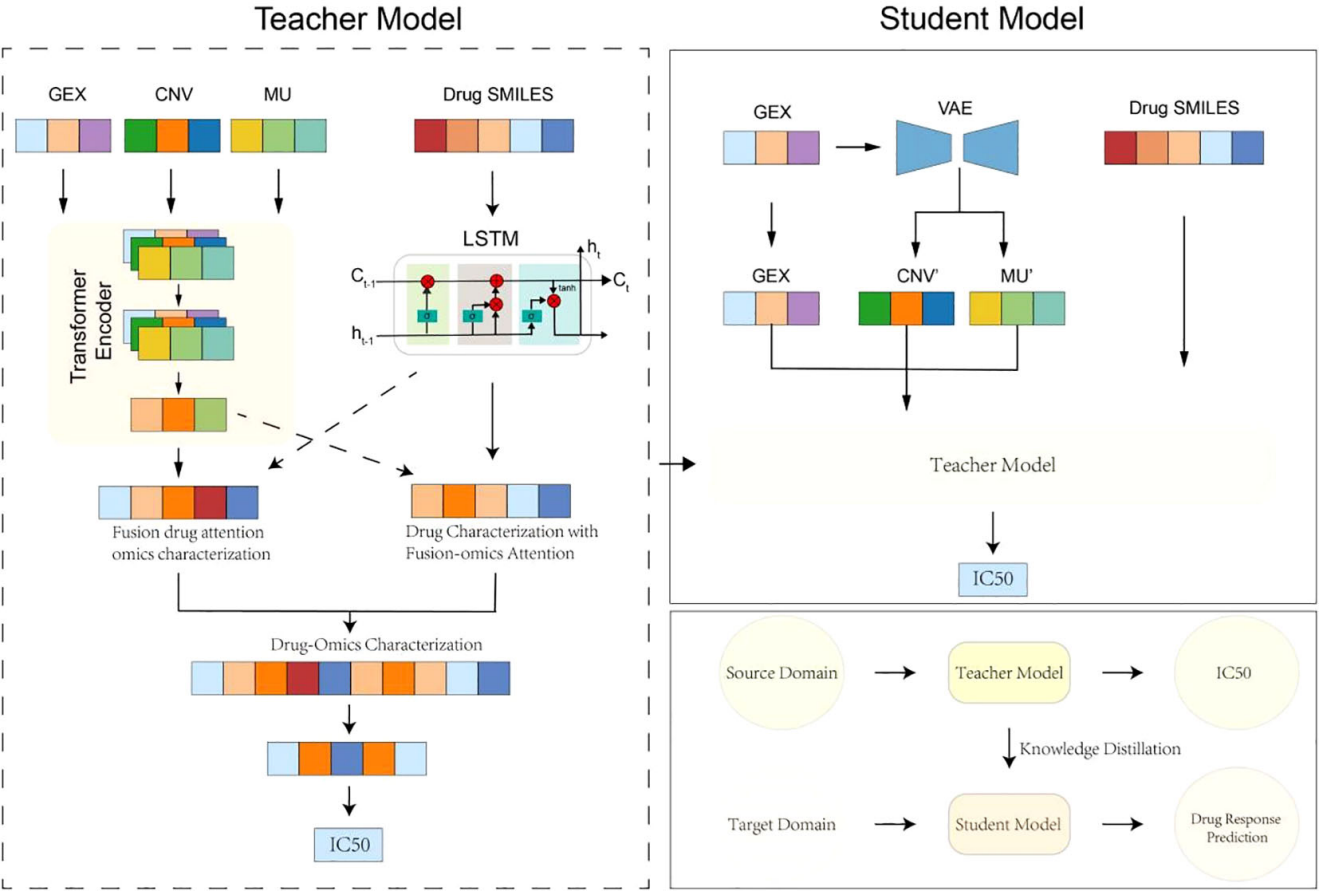
Overview of MKDR framework.

To address the challenge of incomplete omics data in clinical drug response prediction, we propose MKDR (Multi-Omics Modality Completion and Knowledge Distillation for Drug Response Prediction), an end-to-end deep learning framework designed to unify preclinical and clinical prediction tasks. MKDR comprises five key modules: (1) a Transformer-based encoder for multi-omics features, (2) an LSTM-based drug encoder for SMILES representations, (3) a cross-modality attention fusion module, (4) a VAE-based modality completion module, and (5) a knowledge distillation module that enables robust student model training under missing-modality scenarios. As illustrated in **Figure 1**, MKDR integrates these components to extract meaningful drug-cell line representations, complete missing data, and transfer knowledge from complete to incomplete settings, enabling accurate and deployable drug response prediction in cervical cancer.

### Multi-omics feature encoder

To capture the molecular characteristics of cervical cancer, we employ three Transformer encoders to process gene expression (GE), copy number variation (CNV), and mutation (MU) data (30). Each encoder captures long-range dependencies within its modality through stacked self-attention layers:


$$E_{GE}={\text{Transformer}}_{GE}\left(X_{GE}\right)$$

$$E_{CNV}={\text{Transformer}}_{CNV}\left(X_{CNV}\right)$$

$$E_{MU}={\text{Transformer}}_{MU}\left(X_{MU}\right)$$

where $E_{GE}$ , $E_{CNV}$ , $E_{MU}$ denote the high-dimensional embeddings of the genomic, CNV, and mutation data, respectively. The extracted representations provide a comprehensive molecular view of the sample and serve as input to the multi-modal fusion stage. Each encoder uses multi-head self-attention to compute attention weights as:


$$\text{Attention}\left(Q,\;K,\;V\right)=\text{softmax}\left(\frac{QK^{T}}{\sqrt{d}}\right)V$$

where

- Q=W_Q_X (queries),
- K=W_K_X (keys),
- V=W_V_X (values),
- ddd is the feature dimension,
- W_Q_, W_K_, W_V_ are learnable projection matrices.

This mechanism enables the model to learn key gene interactions in a data-driven manner, without relying on predefined biological networks.

### Drug structure encoder

To represent drug molecules at a structural level, we encode canonical SMILES strings using a sequence-based LSTM architecture (31). Each SMILES sequence $\text{S}=s_{1},\;s_{2},\;\cdot \cdot \cdot ,\;s_{n}]$ , where si a tokenized atom or substructure, is embedded into a learnable space and passed through a bi-directional LSTM:


$$H_{drug}=\text{LSTM}\left(E\left(S\right)\right)$$

Here, E(S) denotes the embedded token sequence, and $H_{drug}$ is the final molecular representation derived by mean-pooling or the last hidden state of the LSTM. This module captures sequential dependencies in chemical structure, enabling the model to distinguish subtle molecular differences affecting drug response.

### Cross-modality fusion module

After obtaining representations from the multi-omics and drug encoders, we fuse them using a cross-modality attention mechanism. In this module, omics features serve as keys and values, while the drug representation acts as the query:


$$Z=\text{Attention}\left(\text{Q}={\text{H}}_{\text{drug}},\text{K}=\left[{\text{H}}_{\text{gex}};{\text{H}}_{\text{cnv}};{\text{H}}_{\text{mu}}],\text{ V}=[{\text{H}}_{\text{gex}};{\text{H}}_{\text{cnv}};{\text{H}}_{\text{mu}}\right]\right)$$

This design allows the model to focus on the most drug-relevant molecular signals in the cell line, effectively bridging the gap between compound structure and cellular response. The fused representation Z is passed to the regression layer for IC_50_ prediction.

### Modality completion via VAE

To address missing omics modalities in real-world clinical datasets, we employ a Variational Autoencoder (VAE) to complete absent omics features. The VAE takes observed gene expression $X_{gex}$ ​ as input and reconstructs the missing CNV and mutation vectors:


$$\text{z}∼q_{\phi }(z|X_{gex}),\;X{'}_{cnv},X{'}_{mu}=p_{\theta }(X|z)$$

The VAE is trained by minimizing the standard variational loss:


$$L_{VAE}=E_{q_{\phi }(z|X)}[logp_{\theta }(X|z)-D_{KL}(q_{\phi }(z|X)||p\left(z\right))]$$

This enables robust inference of missing omics under incomplete conditions, improving the student model’s representation quality and downstream performance. We quantitatively evaluated the reconstruction accuracy using PCC, R², MSE, MAE, and AUROC (**Supplementary Table S1**), and further analyzed the effect of KD temperature on imputation performance (**Supplementary Figure S1**).

### Knowledge distillation module

To transfer knowledge from preclinical models trained on complete omics to clinically realistic settings with partial inputs, we adopt a teacher-student knowledge distillation framework. The teacher model is trained with full omics and learns a high-capacity representation for IC_50_ prediction. The student model, which operates on incomplete inputs (e.g., GEX only), is guided by the teacher’s output via distillation loss:


$$L_{KD}=\alpha \cdot L_{task}+\left(1-\alpha \right)\cdot KL(P_{teacher}||P_{student})$$

Here, $L_{task}$ is the standard prediction loss, and KL divergence ensures that the student mimics the teacher’s softened predictions. This mechanism enhances the student model’s robustness in the presence of missing data while maintaining lightweight deployment capacity.

### Evaluation metrics

To comprehensively evaluate the performance of drug response prediction models, we adopted six widely used metrics: mean squared error (MSE), root mean squared error (RMSE), mean absolute error (MAE), coefficient of determination (R²), Pearson correlation coefficient (PCC), and Spearman correlation coefficient (SCC).

- MSE and RMSE measure the average squared and root-squared deviation between predicted and true IC_50_ values, respectively, reflecting both accuracy and outlier sensitivity.
- MAE calculates the average magnitude of prediction errors regardless of direction, providing an interpretable measure of overall error.
- R² evaluates the proportion of variance in the true IC_50_ values explained by the predicted values, indicating the model’s explanatory power.
- PCC and SCC assess the linear and rank-order correlation between predicted and observed responses, respectively, and are especially important for preserving the biological relevance of response patterns across samples.

All metrics were computed on the held-out test set, and lower values for MSE, RMSE, and MAE, along with higher values for R², PCC, and SCC, indicate better model performance.

### Data splitting strategy

Following prior studies on drug sensitivity prediction using large-scale pharmacogenomic datasets such as GDSC and PRISM (1–3), we adopted a stratified data splitting strategy to ensure robust evaluation. Specifically, we randomly divided the drug–cell line pairs into training (80%), validation (10%), and test (10%) subsets, maintaining consistent drug and cell line distributions across each split.

To simulate clinical data limitations and evaluate model robustness under low-resource conditions, we also constructed subsampled training sets with decreasing proportions of the full training data (1/16, 1/8, 1/4, 1/2), while keeping the validation and test sets unchanged. All experiments were repeated with fixed random seeds to ensure reproducibility.

## Result

### Performance evaluation of drug response prediction models

We compared several mainstream models on cervical cancer cell lines using the PRISM dataset, focusing on their accuracy, stability, and generalization across different sample sizes. We evaluated several traditional machine learning methods (Lasso, SVR, RF), deep learning-based models (DeepCDR (7), GADRP (6), BANDRP (9)), and our proposed MKDR framework. We also compared the performance of the MKDR teacher model (with full-modal input) and the student model (with modality-completed input) to further assess MKDR adaptability in modality-missing scenarios.

**Figure 2:**
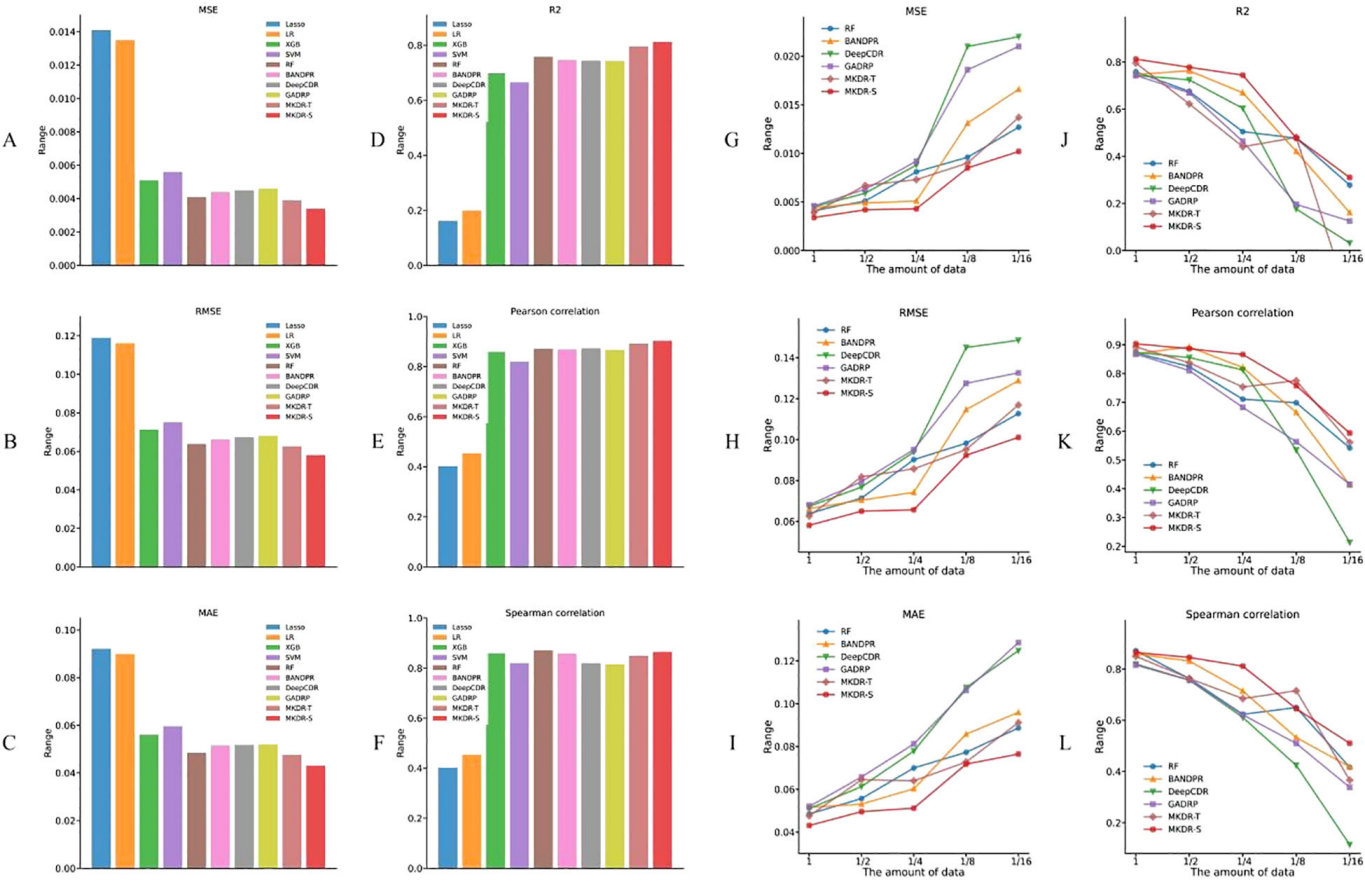
Drug response prediction accuracy of MKDR and baseline models on cervical cancer cell lines. **(A–F)** Performance comparison under full training data across six metrics (MSE, RMSE, MAE, R², PCC, SCC), showing MKDR-Student and MKDR-Teacher outperforming all baseline models. **(G–L)** Model robustness under reduced training sizes (1/16 to full), where MKDR-Student consistently maintains superior performance across all metrics compared to traditional and deep learning methods.

With full training data, both MKDR-Student and MKDR-Teacher outperformed traditional machine learning and deep learning models across six metrics (MSE, RMSE, MAE, R², PCC, and SCC) (**Figures 2A–F**). For error-based metrics, MKDR-Student achieved an MSE of only 0.0034, representing a ~17% reduction compared to Random Forest (RF, 0.0041), and showing clear advantages over Lasso (0.0141), SVR (0.0056), and deep models such as GADRP (0.0046), DeepCDR (0.0045), and BANDRP (0.0044) (**Figure 2A**, **Table 1**). For RMSE and MAE, MKDR-Student achieved the lowest values of 0.0581 and 0.0431, respectively (**Figures 2B, C**), confirming its strength in minimizing numerical errors. For correlation-based metrics, MKDR-Student also stood out with an R² of 0.8126, surpassing RF (0.7588), GADRP (0.7435), BANDRP (0.7475), and DeepCDR (0.7440) (**Figure 2D**, **Table 1**), reflecting its superior fit. MKDR-Student also achieved the highest PCC (0.9033) and SCC (0.8647) (**Figures 2E, F**), indicating both high prediction accuracy and stronger biological interpretability via consistent sample ranking.

**Table 1:** Performance comparison of different models for drug response prediction on cervical cancer cell lines (PRISM dataset).

Methods	Dataset Split	MSE	RMSE	MAE	R2	PCC	SCC
XGB	5-fold	0.0051	0.0713	0.0561	0.6987	0.8592	0.8592
SVM	5-fold	0.0056	0.0751	0.0596	0.6657	0.8200	0.8200
RF	5-fold	0.0041	0.0638	0.0485	0.7588	0.8712	0.8712
LR	5-fold	0.0135	0.1162	0.0899	0.1998	0.4541	0.4541
Lasso	5-fold	0.0141	0.1189	0.0921	0.1618	0.4023	0.4023
BANDPR	5-fold	0.0044	0.0663	0.0516	0.7475	0.8690	0.8587
DeepCDR	5-fold	0.0045	0.0673	0.0510	0.7440	0.8732	0.8197
GADRP	5-fold	0.0046	0.0681	0.052	0.7435	0.8672	0.8153
MKDR-Teacher	5-fold	0.0039	0.0626	0.0476	0.7957	0.8922	0.8495
MKDR-Student	5-fold	**0.0034**	**0.0581**	**0.0431**	**0.8126**	**0.9033**	**0.8647**

The bold values highlight the accuracy of the student model in MKDR.

To mimic data scarcity common in real-world clinical studies, we constructed training subsets ranging from 1/16 to the full dataset and evaluated model performance across six metrics (**Figures 2G–L**). Most baseline models experienced sharp degradation as data volume decreased, with DeepCDR and BANDRP showing substantial fluctuations due to their dependence on complete multi-omics inputs and large training sets. In contrast, MKDR-Student maintained stable error and correlation metrics across all sample sizes, highlighting its robustness to data scarcity. Under the extreme 1/16 condition, MKDR-Student preserved an R² > 0.51, while Lasso, SVR, and other models dropped to near-zero. It also outperformed all baselines at each scale, achieving 40–50% lower MSE and RMSE than BANDRP at 1/8 data size. This robustness stems from MKDR’s two key components: a VAE-based modality completion module that infers missing omics features from gene expression, and a knowledge distillation module that transfers learned representations from a full-modality teacher model. Interestingly, MKDR-Student occasionally outperforms the Teacher model. This may be due to the student’s exposure to a wide range of incomplete modality scenarios during training, which encourages the learning of more generalizable and robust representations. In contrast, the Teacher model is optimized for full-modality inputs and may be more prone to overfitting specific feature patterns (32, 33). Together, these mechanisms mitigate overfitting and enhance generalization under limited supervision. MKDR’s resilience and its ability to preserve sample ranking make it particularly suited for clinical deployment, especially in early-stage precision oncology trials where multi-omics data are often sparse or incomplete. To further assess its generalization ability, we additionally validated MKDR-Student on the GDSC SISO cervical cancer cell line and the TCGA-CESC patient cohort. The model achieved a PCC of 0.7682 for IC_50_ prediction on SISO and an AUROC of 0.635 for cisplatin response classification in cisplatin-treated patients (n = 99) on TCGA-CESC, demonstrating robust performance across both preclinical and clinical settings (**Supplementary Tables S2**, **S3**).

### Variations in the drug response prediction landscape and accuracy differences across different drugs

To understand the representational advantages of MKDR, we analyzed the evolution of its learned features in comparison to BANDRP, a strong and stable baseline known for its multimodal encoding capabilities. To analyze the evolution of representation learning, we extracted latent features from three stages of each model: (1) initial omics and drug embeddings, (2) fused representations after multimodal integration, and (3) final latent vectors used for IC_50_ prediction. In addition to visualizing these latent spaces, we compared the MSE of MKDR (teacher and student) with BANDRP across several common anticancer drugs (e.g., Methotrexate, Pemetrexed) and clinically relevant cervical cancer treatments (e.g., Topotecan, Paclitaxel, Docetaxel (34).

**Figure 3:**
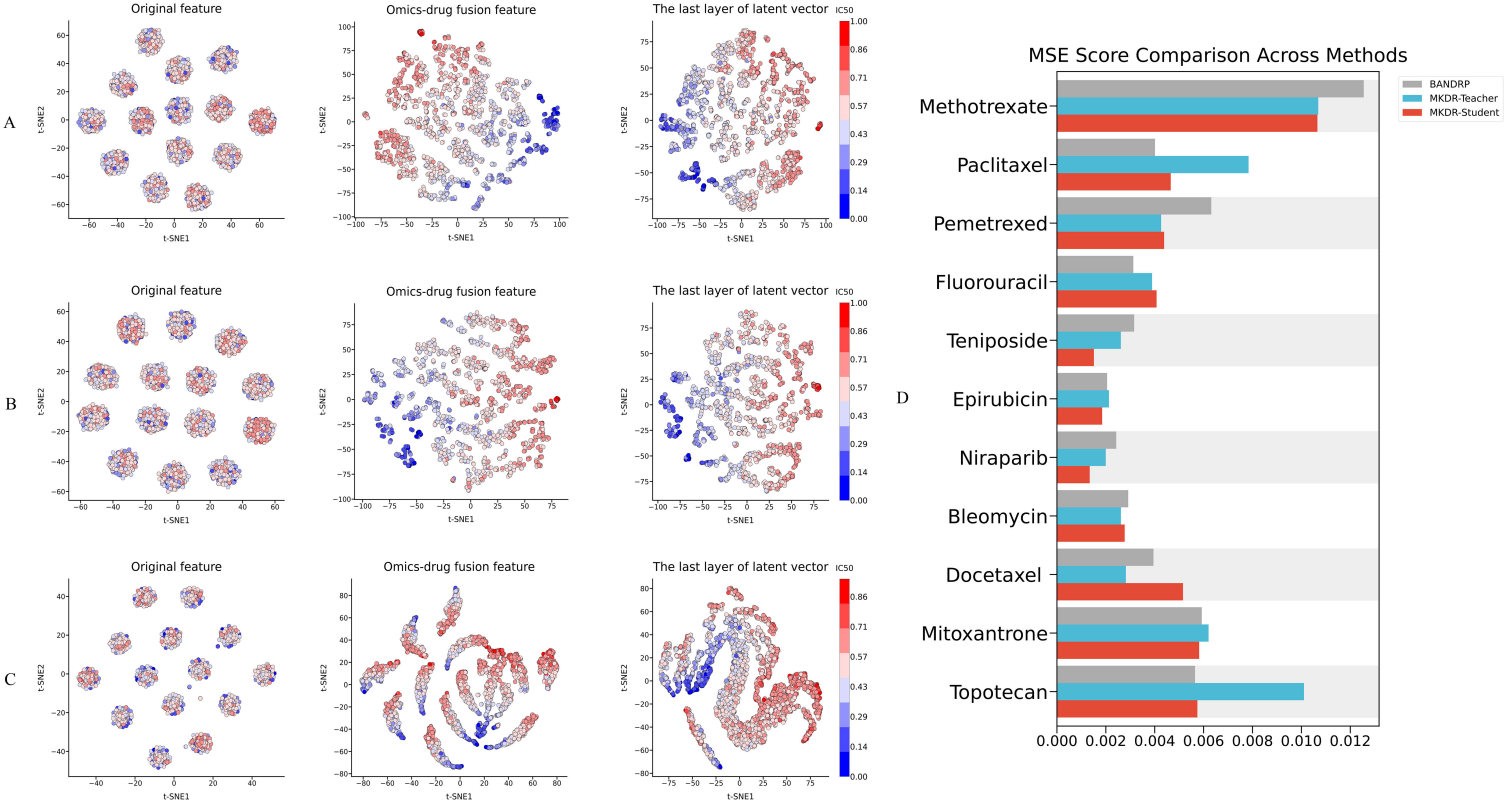
Evolution of the drug response prediction space and accuracy comparison across drugs in different models. **(A–C)** Visualization of feature evolution in BANDRP **(A)**, MKDR-Teacher **(B)**, and MKDR-Student **(C)**, including original features, omics–drug fusion features, and the final latent vectors used for prediction. Samples are colored by IC_50_ values to reflect model alignment with drug sensitivity gradients. **(D)** MSE comparison across cervical cancer-related and commonly used anticancer drugs, showing that both MKDR variants consistently outperform BANDRP, especially on clinically relevant treatments such as Topotecan, Paclitaxel, and Docetaxel.

We visualized the evolution of learned representations in MKDR-Teacher (**Figure 3A**), MKDR-Student (**Figure 3B**), and BANDRP (**Figure 3C**) to assess their ability to encode and interpret drug response. In the raw feature space (left column), all models showed entangled distributions with no clear separation between sensitive and non-sensitive samples, indicating unextracted biological signals. After omics–drug fusion (middle column), MKDR models formed distinct clusters, with classes diverging in different directions—suggesting effective integration of biological and chemical information. In contrast, BANDRP retained partial structure, but class overlap remained substantial. In the final latent space for IC_50_ prediction (right column), MKDR-Teacher achieved the sharpest class boundaries, with MKDR-Student closely following, reflecting successful knowledge transfer. These findings confirm that the distillation-based MKDR architecture improves feature separability, interpretability, and robustness—while also supporting model compression and generalization under limited multi-omics conditions (35, 36).

Additionally, bar plots on the right side of **Figure 3** compare IC_50_ prediction MSEs across ten representative anticancer drugs(**Figure 3D**). MKDR-Teacher achieved the lowest error on 7 out of 10 drugs (e.g., 0.0011 for Methotrexate, 0.0023 for Pemetrexed), showing strong cross-drug generalizability. MKDR-Student, though lighter, reached comparable accuracy (e.g., 0.0012 and 0.0027 for the same drugs), confirming efficient knowledge transfer. In contrast, BANDRP exhibited consistently higher errors (e.g., 0.0071 for Paclitaxel, 0.0099 for Topotecan), highlighting its limitations in modeling heterogeneous responses and fusing complex multi-omics features. Together, these results underscore the advantage of MKDR’s distillation strategy in achieving robust, generalizable performance across diverse drug scenarios.

### Ablation study and robustness evaluation of the MKDR model

**Figure 4:**
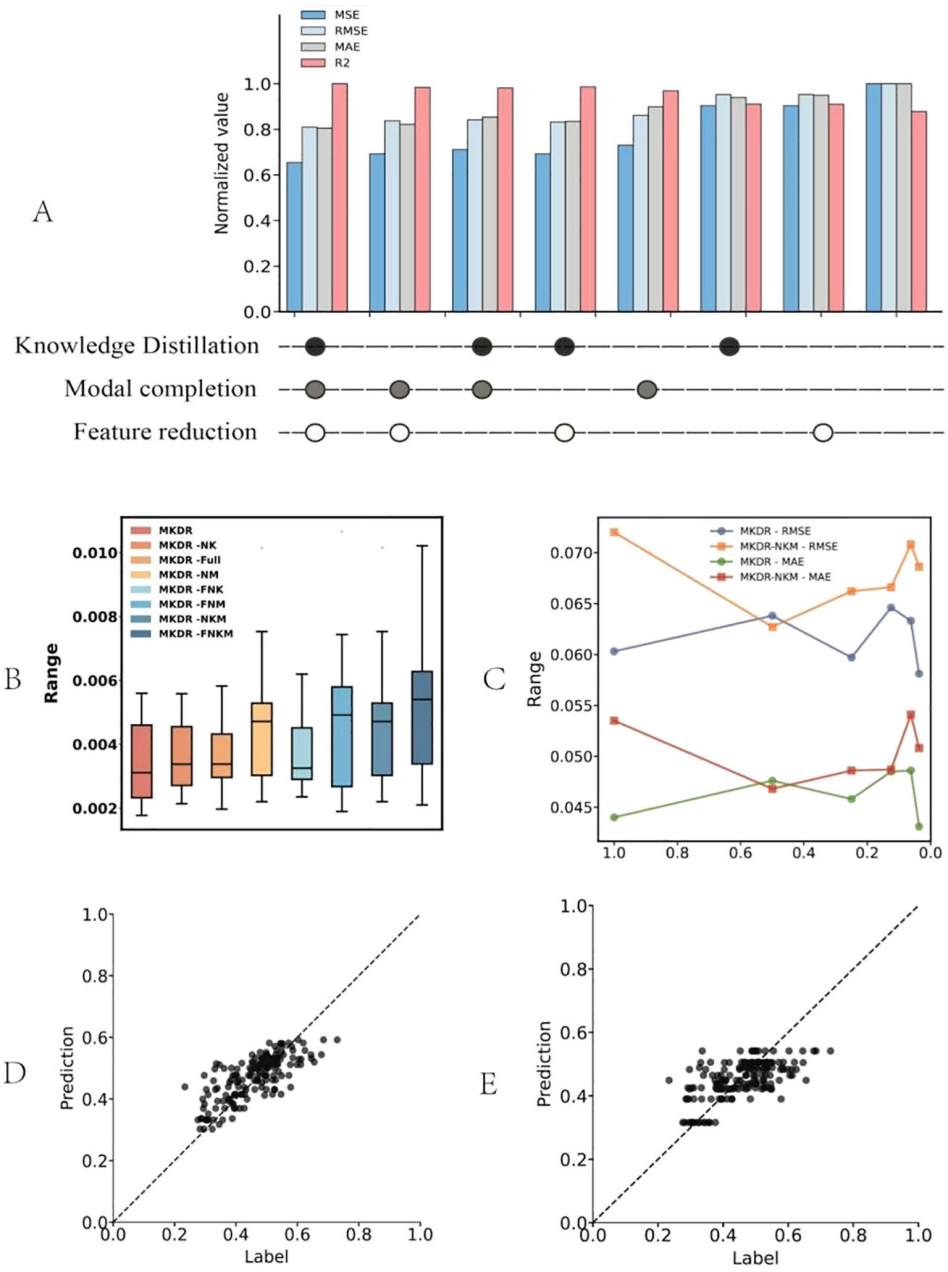
Ablation study and robustness evaluation results of the MKDR model. **(A)** Ablation of core modules (knowledge distillation, modality completion, feature reduction) shows that all components contribute to optimal performance, with the full model achieving the best metrics. **(B)** Error distribution across ablated variants indicates that removing modality completion or distillation increases variance, while the full model remains stable. **(C)** Performance under different feature compression levels shows MKDR retains stability even at 20% dimensionality, unlike variants lacking core modules. **(D, E)** Regression fits show MKDR outputs align well with true labels, while MKDR-NKM collapses toward the center and fails to capture extremes.

To further validate MKDR’s performance, we conducted ablation studies to quantify the contributions of its core components—knowledge distillation, modality completion, and feature compression—to drug response prediction. These modules collectively enhanced model adaptability and stability under complex multi-omics conditions (**Figure 4A**).

We first evaluated the contributions of different MKDR modules—knowledge distillation, modality completion, and feature compression—through ablation studies (**Figure 4A**). The full model achieved the best performance across all normalized metrics, with R² close to 1 (0.98), and low MSE (0.24), RMSE (0.34), and MAE (0.29), demonstrating strong predictive accuracy. Removing any module led to a systematic decline, confirming their complementary effects. Notably, excluding knowledge distillation (MKDR-NK) resulted in the largest performance drop (MSE up to 0.295, ~23% higher), underscoring the teacher model’s critical role in feature transfer. In contrast, modality completion contributed more to robustness: even without distillation, the model retained high R² (~0.92) and low MAE (~0.33), outperforming the distillation-only variant. This supports the view that handling missing omics data directly is more effective for stability than transfer-based learning alone—consistent with the consensus in multi-modal learning that “missing information is the primary source of perturbation” (37).

We further evaluated MKDR’s prediction stability and robustness under different module settings and compression levels. As shown in **Figure 4B**, the full MKDR model exhibited the most compact error distribution (median MSE ~0.0034), while removing knowledge distillation (MKDR-NK) or modality completion (MKDR-NM) increased variance. The MKDR-FNKM variant showed the widest error range (~0.009), indicating severe instability. Among all modules, modality completion played a greater role in controlling sample-wise variance, while distillation enhanced output consistency. Under feature compression (**Figure 4C**), MKDR maintained consistent performance down to 20% of its original feature dimension (RMSE < 0.062, MAE < 0.045). Slight performance gains at 80–60% compression suggest improved signal-to-noise ratio via redundant feature removal. In contrast, MKDR-NKM degraded rapidly below 40% (RMSE ~0.071, MAE > 0.050), accompanied by large fluctuations—revealing poor generalization under constrained representations. Regression fitting (**Figures 4D, E**) confirmed these trends. MKDR outputs aligned well with true labels across the full response range, showing high sensitivity and diagonal consistency. In contrast, MKDR-NKM exhibited response collapse, with 83% of predictions concentrated in the IC_50_ 0.3–0.7 μM range and a 2.1-fold increase in low-response deviation (p = 0.012), impairing the model’s ability to capture extremes. These issues align with earlier findings on increased variance and compression sensitivity.

Overall, these results highlight the complementary roles of knowledge distillation, modality completion, and feature compression in enhancing MKDR’s stability, compression tolerance, and clinical discrimination capability. This modular design supports deployment in real-world multi-omics scenarios and precision medicine under resource-limited conditions.

### Interpretable drug response prediction with teacher and student models

To evaluate the interpretability of MKDR in multi-omics integration, we analyzed input feature importance using the Integrated Gradients method. This technique quantifies each feature’s contribution to the prediction outcome, enabling insight into the roles of different omics modalities. We compared the feature attribution patterns of the teacher and student models to assess whether knowledge distillation and modality completion allow the student to recover biologically meaningful representations. This analysis also helps identify key omics features that drive drug response prediction.

**Figure 5:**
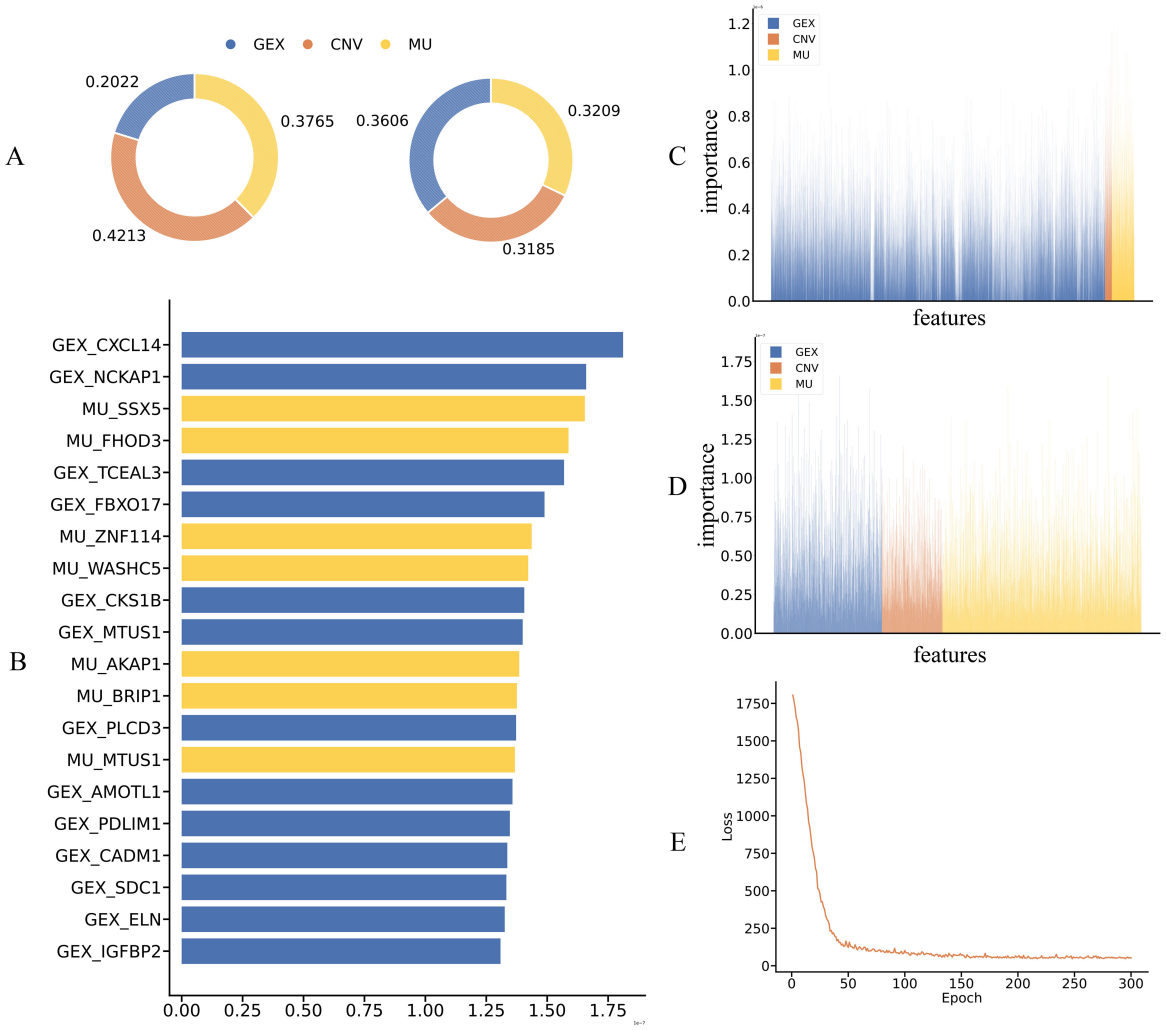
Interpretability analysis and key feature contributions of the teacher and student models. **(A)** Distribution of input feature contributions across omics modalities (GEX, CNV, MU) for teacher and student models, showing modality-level attribution alignment. **(B)** Top 20 most influential features ranked by integrated gradient scores, highlighting consistent GEX and MU contributions across both models. **(C, D)** Feature attribution heatmaps from teacher **(C)** and student **(D)** models, demonstrating consistent modality-aware importance and successful transfer of interpretability via distillation and completion. **(E)** Training loss curve of the teacher model, confirming convergence and stability.

We first compared the reliance on different omics modalities between the teacher and student models. In the teacher model with full multi-omics input, gene expression (GEX) contributed the most to prediction (42.13%), while CNV and mutation accounted for 20.22% and 37.65%, respectively—indicating a preference for transcriptomic signals. In contrast, the student model, despite receiving only GEX input, achieved a balanced attribution across modalities through modality completion and knowledge distillation, with feature importance at 36.06% (GEX), 31.85% (CNV), and 32.09% (mutation).This shift from expression dominance to multi-modal synergy suggests that the student effectively inherits the teacher’s semantic structure and reconstructs missing modality representations, enabling biologically meaningful integration and improved generalization. Feature importance distributions are shown in **Figure 5A**.

We further analyzed the top 20 most important features in the MKDR-Student model to assess whether multi-omics knowledge was effectively learned. Among them, 7 features originated from the mutation (MU) modality, highlighting the relevance of mutation signals in predicting cervical cancer drug response. This aligns with prior studies linking mutation-driven mechanisms to cervical cancer. For example, CXCL14 is frequently silenced by promoter hypermethylation induced by HPV E7 oncoproteins, weakening antitumor immune responses (38, 39). Its loss also downregulates MHC-I and impairs CD8^+^ T cell activation, promoting immune evasion. Another top-ranked feature, MTUS1, acts via the PTENP1/miR-19b/MTUS1 axis to suppress proliferation and invasion in cervical cancer and is often downregulated in aggressive tumors (40).

To verify whether the student model reconstructs multi-omics information, we visualized the full distribution of feature importance derived from Integrated Gradients. As shown in **Figures 5C, D**, the student model’s importance scores across GEX, CNV, and MU resemble those of the teacher model, despite being trained only on GEX. This close alignment indicates that modality completion enables reliable inference from missing inputs. Notably, the student model’s emphasis on mutation features correlates strongly with the high MU ranking observed in **Figure 5B**, confirming that mutation-derived signals were indeed used in prediction. Additionally, the training curve of the modality completion module (**Figure 5E**) shows a rapid loss decrease within the first 100 epochs followed by convergence, indicating successful reconstruction and a stable foundation for downstream representation learning.

In conclusion, MKDR effectively internalizes multi-omics knowledge even with incomplete inputs. By integrating modality completion and knowledge distillation, it assigns meaningful importance to both expression and mutation features, achieving structural generalization and mechanistic recovery in drug response prediction. These results reinforce MKDR’s promise for robust, interpretable multi-omics modeling.

While MKDR has demonstrated robust performance across various evaluations, certain limitations should be acknowledged in the context of our study scope. This work specifically focuses on cervical cancer cell lines to assess the feasibility of multi-omics integration under missing-modality conditions. Accordingly, the dataset consists of 15 well-characterized cell lines, which may influence generalizability to broader clinical populations. Although the model performs well under simulated real-world scenarios, future validation using patient-derived datasets and prospective clinical data would further substantiate its translational relevance. These directions extend beyond the scope of the current study but represent important avenues for future investigation.

## Discussion

In this study, we proposed MKDR, a multi-omics framework that demonstrates strong predictive performance, robustness, and interpretability under incomplete data conditions in cervical cancer. By integrating modality completion and knowledge distillation, MKDR maintains high accuracy not only with complete inputs but also under scenarios of missing modalities and feature compression, offering a new paradigm for precision medicine under multi-modal data scarcity.

First, in overall performance evaluation, the MKDR-Student model consistently outperformed traditional machine learning and deep learning baselines across MSE, MAE, and R² metrics—and even slightly surpassed the teacher model. In simulated clinical scenarios lacking certain omics inputs, the student model effectively reproduced full-modality representations via completion and distillation. MKDR also showed strong generalization across individual drugs: for 7 out of 10 clinically relevant anticancer compounds (e.g., Topotecan, Paclitaxel, Pemetrexed), the teacher model achieved the lowest prediction error, with the student closely matching its accuracy. These results suggest that MKDR is not overly dependent on input completeness or specific signal structures and thus generalizes well across heterogeneous drug–feature spaces. To further evaluate the clinical applicability of MKDR, we validated its predictions on TCGA-CESC patients by assessing the association between predicted drug sensitivity scores and treatment response categories derived from RECIST annotations. While IC_50_ values provide important insights into drug potency in preclinical settings, they may not fully capture therapeutic efficacy *in vivo* due to the lack of immune–tumor interactions, stromal context, and inter-patient variability (41). The ability of MKDR to transfer learned representations from cell lines to patients suggests that its latent space may capture biologically conserved response features, offering a promising route to bridge preclinical–clinical discrepancies in drug response prediction. Although the current study focuses on drug response prediction across cervical cancer as a whole, we acknowledge that future investigations should explore model performance across HPV subtypes (e.g., HPV16/18) and histological subtypes (e.g., squamous *vs*. adenocarcinoma), as these factors may influence molecular profiles and treatment sensitivity. We also suggest incorporating *in vivo* assays or patient-derived xenograft models in future studies to complement our in silico findings.

Beyond performance, MKDR’s representational trajectory reveals deeper modeling advantages. In a three-stage latent space comparison, the student model, starting from GEX-only input, gradually aligns with the teacher’s fused embedding and ultimately forms clearly separable clusters in the predictive space. This structural convergence validates the synergy between modality completion and distillation and highlights the model’s ability to extract meaningful biological signals within a nonlinear embedding space.

MKDR also exhibits strong interpretability. Feature attribution analysis via integrated gradients revealed that key mutation-derived features (e.g., CXCL14, MTUS1) received high importance scores in the student model. Literature evidence supports their biological relevance: CXCL14 is silenced via HPV E7-induced promoter hypermethylation (38), impairing immune recognition, while MTUS1 functions as a tumor suppressor and is downregulated in invasive cervical cancer (40). These findings validate the plausibility of MKDR’s reconstructed omics signals and point to its potential in mechanism discovery and biomarker identification. These findings suggest that MKDR not only identifies predictive features but also captures biologically meaningful associations aligned with known drug mechanisms. Notably, MKDR shows high accuracy for key drugs like Topotecan and Paclitaxel (**Figure 3D**). For Topotecan, Integrated Gradients highlight DNA repair and cell cycle genes, aligning with its known mechanism of inducing replication-related DNA breaks through topoisomerase I inhibition (42).

In summary, MKDR achieves accurate drug response prediction while addressing three core challenges: incomplete omics, feature compression, and biological interpretability. Its modular architecture and cross-modal reasoning make it well suited for large-scale drug screening, translational modeling—demonstrating the practical utility of multi-modal AI frameworks in precision oncology.

## Conclusion

In this study, we proposed the MKDR framework for drug response prediction, which integrates modality completion and knowledge distillation into a unified learning model designed to address scenarios with incomplete multi-omics information. In modeling drug responses for cervical cancer, MKDR achieved state-of-the-art performance in terms of prediction accuracy, model stability, structural compression compatibility, and feature-level interpretability. The model demonstrated excellent generalization and practical applicability across settings with missing modalities, low-dimensional features, and diverse drug backgrounds, making it particularly suitable for real-world clinical data characterized by sparsity and limited sample size.

Future work may further extend MKDR to other cancer types and incorporate additional omics modalities (such as proteomics and spatial transcriptomics), along with validation using translational medicine datasets for patient-level prediction, supporting more actionable individualized treatment recommendations. Moreover, as current evaluations are limited to cervical cancer, the scalability and effectiveness of MKDR on larger drug response datasets and in clinical applications warrant further investigation through extensive experimental validation.

## Data Availability

The multi-omics data used in this study were obtained from publicly available sources. Omics profiles were downloaded from the Cancer Cell Line Encyclopedia (https://portals.broadinstitute.org/ccle), and drug response data were retrieved from the PRISM Repurposing dataset (https://depmap.org/portal/prism/). A compiled version of the data used in this study is available at https://github.com/bowei-color/MKDR/tree/main/data/source.

## References

[1] Sung H Ferlay J Siegel RL Laversanne M Soerjomataram I Jemal A. Global cancer statistics 2020: GLOBOCAN estimates of incidence and mortality worldwide for 36 cancers in 185 countries. CA Cancer J Clin. (2021) 71:209–49. doi: 10.3322/caac.21660, PMID: 33538338

[2] Pak M Lee S Sung I Koo B Kim S. Improved drug response prediction by drug target data integration via network-based profiling. Brief Bioinform. (2023) 24. doi: 10.1093/bib/bbad034, PMID: 36752352

[3] Zhu J Wang J Wang X Gao M Guo B Gao M. Prediction of drug efficacy from transcriptional profiles with deep learning. Nat Biotechnol. (2021) 39:1444–52. doi: 10.1038/s41587-021-00946-z, PMID: 34140681

[4] Chen C Wang J Pan D Wang X Xu Y Yan J. Applications of multi-omics analysis in human diseases. MedComm (2020). (2023) 4:e315. doi: 10.1002/mco2.315, PMID: 37533767 PMC10390758

[5] Lao C Zheng P Chen H Liu Q An F Li Z. DeepAEG: a model for predicting cancer drug response based on data enhancement and edge-collaborative update strategies. BMC Bioinf. (2024) 25:105. doi: 10.1186/s12859-024-05723-8, PMID: 38461284 PMC10925015

[6] Wang H Dai C Wen Y Wang X Liu W He S. GADRP: graph convolutional networks and autoencoders for cancer drug response prediction. Brief Bioinform. (2023) 24. doi: 10.1093/bib/bbac501, PMID: 36460622

[7] Liu Q Hu Z Jiang R Zhou M. DeepCDR: a hybrid graph convolutional network for predicting cancer drug response. Bioinformatics. (2020) 36:i911–8. doi: 10.1093/bioinformatics/btaa822, PMID: 33381841

[8] Sharifi-Noghabi H Zolotareva O Collins CC Ester M. MOLI: multi-omics late integration with deep neural networks for drug response prediction. Bioinformatics. (2019) 35:i501–9. doi: 10.1093/bioinformatics/btz318, PMID: 31510700 PMC6612815

[9] Cao C Zhao H Wang J. BANDRP: a bilinear attention network for anti-cancer drug response prediction based on fingerprint and multi-omics. Brief Bioinform. (2024) 25. doi: 10.1093/bib/bbae493, PMID: 39406520 PMC11479717

[10] Vitorino R. Transforming clinical research: the power of high-throughput omics integration. Proteomes. (2024) 12. doi: 10.3390/proteomes12030025, PMID: 39311198 PMC11417901

[11] Subramanian I Verma S Kumar S Jere A Anamika K. Multi-omics data integration, interpretation, and its application. Bioinform Biol Insights. (2020) 14:1177932219899051. doi: 10.1177/1177932219899051, PMID: 32076369 PMC7003173

[12] Multi-omics prognostic marker discovery and survival modeling: A case study on pan-cancer survival analyses in women’s cancers. doi: 10.1101/2025.01.08.25320212 PMC12540658 41120506

[13] Flores JE Claborne DM Weller ZD Webb-Robertson BM Waters KM Bramer LM. Missing data in multi-omics integration: Recent advances through artificial intelligence. Front Artif Intell. (2023) 6:1098308. doi: 10.3389/frai.2023.1098308, PMID: 36844425 PMC9949722

[14] Daly DS Anderson KK Panisko EA Purvine SO Fang R Monroe ME. Mixed-effects statistical model for comparative LC-MS proteomics studies. J Proteome Res. (2008) 7:1209–17. doi: 10.1021/pr070441i, PMID: 18251496

[15] Webb-Robertson BJ Wiberg HK Matzke MM Brown JN Wang J McDermott JE. Review, evaluation, and discussion of the challenges of missing value imputation for mass spectrometry-based label-free global proteomics. J Proteome Res. (2015) 14:1993–2001. doi: 10.1021/pr501138h, PMID: 25855118 PMC4776766

[16] Brenes A Hukelmann J Bensaddek D Lamond AI. Multibatch TMT reveals false positives, batch effects and missing values. Mol Cell Proteomics. (2019) 18:1967–80. doi: 10.1074/mcp.RA119.001472, PMID: 31332098 PMC6773557

[17] Adam G Rampasek L Safikhani Z Smirnov P Haibe-Kains B Goldenberg A. Machine learning approaches to drug response prediction: challenges and recent progress. NPJ Precis Oncol. (2020) 4:19. doi: 10.1038/s41698-020-0122-1, PMID: 32566759 PMC7296033

[18] Rashid MM Selvarajoo K. Advancing drug-response prediction using multi-modal and -omics machine learning integration (MOMLIN): a case study on breast cancer clinical data. Brief Bioinform. (2024) 25. doi: 10.1093/bib/bbae300, PMID: 38904542 PMC11190965

[19] Gao H Korn JM Ferretti S Monahan JE Wang Y Singh M. High-throughput screening using patient-derived tumor xenografts to predict clinical trial drug response. Nat Med. (2015) 21:1318–25. doi: 10.1038/nm.3954, PMID: 26479923

[20] Zhang S Xue Z. Progress of epigenetic methylation in lung cancer research. Zhongguo Fei Ai Za Zhi. (2017) 20:635–40. doi: 10.3779/j.issn.1009-3419.2017.09.08, PMID: 28935018 PMC5973367

[21] Zitnik M Nguyen F Wang B Leskovec J Goldenberg A Hoffman MM. Machine learning for integrating data in biology and medicine: principles, practice, and opportunities. Inf Fusion. (2019) 50:71–91. doi: 10.1016/j.inffus.2018.09.012, PMID: 30467459 PMC6242341

[22] Hinton G Vinyals O Dean J. Distilling the knowledge in a neural network. arXiv preprint arXiv:1503.02531. (2015). doi: 10.48550/arXiv.1503.02531

[23] Ahn H Xie S Xing E. (2019). Collaborative learning for multi-task learning via knowledge distillation, in: Proceedings of the IEEE/CVF Conference on Computer Vision and Pattern Recognition (CVPR), . pp. 6567–76.

[24] He Z Hu S Chen Y An S Zhou J Liu R. Mosaic integration and knowledge transfer of single-cell multimodal data with MIDAS. Nat Biotechnol. (2024) 42:1594–605. doi: 10.1038/s41587-023-02040-y, PMID: 38263515 PMC11471558

[25] Zhang X Zhao Y Wang J Wang Y Xu M. (2021). Cross-modal knowledge distillation with meta learning for multimodal learning, in: Proceedings of the 29th ACM International Conference on Multimedia, . pp. 3445–53.

[26] Barretina J Caponigro G Stransky N Venkatesan K Margolin AA Kim S. The Cancer Cell Line Encyclopedia enables predictive modelling of anticancer drug sensitivity. Nature. (2012) 483:603–7. doi: 10.1038/nature11003, PMID: 22460905 PMC3320027

[27] Corsello SM Nagari RT Spangler RD Rossen J Kocak M Bryan JG. Discovering the anti-cancer potential of non-oncology drugs by systematic viability profiling. Nat Cancer. (2020) 1:235–48. doi: 10.1038/s43018-019-0018-6, PMID: 32613204 PMC7328899

[28] Yang W Soares J Greninger P Edelman EJ Lightfoot H Forbes S. Genomics of Drug Sensitivity in Cancer (GDSC): a resource for therapeutic biomarker discovery in cancer cells. Nucleic Acids Res. (2013) 41:D955–61. doi: 10.1093/nar/gks1111, PMID: 23180760 PMC3531057

[29] Tomczak K Czerwinska P Wiznerowicz M. The Cancer Genome Atlas (TCGA): an immeasurable source of knowledge. Contemp Oncol (Pozn). (2015) 19:A68–77. doi: 10.5114/wo.2014.47136, PMID: 25691825 PMC4322527

[30] Li G Fu S Wang S Zhu C Duan B Tang C. A deep generative model for multi-view profiling of single-cell RNA-seq and ATAC-seq data. Genome Biol. (2022) 23:20. doi: 10.1186/s13059-021-02595-6, PMID: 35022082 PMC8756637

[31] Loeffler HH He J Tibo A Janet JP Voronov A Mervin LH. Reinvent 4: Modern AI-driven generative molecule design. J Cheminform. (2024) 16:20. doi: 10.1186/s13321-024-00812-5, PMID: 38383444 PMC10882833

[32] Bai T Zhao J Wen B. Guided adversarial contrastive distillation for robust students. IEEE Trans Inf Forensics Secur. (2024) 19:9643–55. doi: 10.1109/TIFS.2023.3237371

[33] Wang J Liao D Zhang Y Xu D Zhang X. Layerwised multimodal knowledge distillation for vision-language pretrained model. Neural Networks. (2024) 175:106272. doi: 10.1016/j.neunet.2024.106272, PMID: 38569460

[34] Markman M. Advances in cervical cancer pharmacotherapies. Expert Rev Clin Pharmacol. (2014) 7:219–23. doi: 10.1586/17512433.2014.884924, PMID: 24490716

[35] Wang T Gong Y Liu J Lu H. Learning from few samples with knowledge distillation for classifying histopathological images. IEEE Access. (2019) 7:172830–9.

[36] Guo J Xu Z Liu Y Zhao L. (2020). Multimodal knowledge distillation for modality alignment and generalization, in: Proceedings of the AAAI Conference on Artificial Intelligence, , Vol. 34. pp. 4010–7.

[37] Das S Mukhopadhyay I. TiMEG: an integrative statistical method for partially missing multi-omics data. Sci Rep. (2021) 11:24077. doi: 10.1038/s41598-021-03034-z, PMID: 34911979 PMC8674330

[38] Cicchini L Westrich JA Xu T Vermeer DW Berger JN Clambey ET. Suppression of antitumor immune responses by human papillomavirus through epigenetic downregulation of CXCL14. mBio. (2016) 7. doi: 10.1128/mBio.00270-16, PMID: 27143385 PMC4959654

[39] Cao B Yang Y Pan Y Jia Y Brock MV Herman JG. Epigenetic silencing of CXCL14 induced colorectal cancer migration and invasion. Discov Med. (2013) 16:137–47., PMID: 24099668 PMC4061567

[40] Ou L Xiang TY Hao XY Wang DZ Zeng Q. Reduced long non-coding RNA PTENP1 contributed to proliferation and invasion via miR-19b/MTUS1 axis in patients with cervical cancer. Eur Rev Med Pharmacol Sci. (2020) 24:4132–44. doi: 10.26355/eurrev_202004_20993, PMID: 32373949

[41] Rahman MM Wells G Rantala JK Helleday T Muthana M Danson SJ. In-vitro assays for immuno-oncology drug efficacy assessment and screening for personalized cancer therapy: scopes and challenges. Expert Rev Clin Immunol. (2024) 20:821–38. doi: 10.1080/1744666X.2024.2336583, PMID: 38546609

[42] Patel AG Flatten KS Peterson KL Beito TG Schneider PA Perkins AL. Immunodetection of human topoisomerase I-DNA covalent complexes. Nucleic Acids Res. (2016) 44:2816–26. doi: 10.1093/nar/gkw109, PMID: 26917015 PMC4824114

