# Supplementary material for: Multi-omics modality completion and knowledge distillation for drug response prediction in cervical cancer
Source: Front Oncol. 2025 Aug 27;15:1622600. doi: 10.3389/fonc.2025.1622600 (PMC12427122; doi:10.3389/fonc.2025.1622600)
Supplement: Supplementary file 1 [file DataSheet1.pdf]

Supplementary Tables and Figures

Table S1. Quantitative evaluation of reconstruction performance for CNV and mutation features using gene expression input. Metrics include Pearson correlation coefficient (PCC), mean squared error (MSE), mean absolute error (MAE), and coefficient of determination (R^2).

Reconstructed Modality	PCC	MSE	MAE	R^2
CNV	0.9939	0.1501	0.1840	0.9657
Mutation	0.9936	0.1520	0.1861	0.9648

We evaluated the reconstruction quality of CNV and mutation features based on gene expression input using a Variational Autoencoder (VAE). Results show high reconstruction fidelity across both modalities, with PCC > 0.99 and R^2 > 0.96, confirming effective modality recovery from gene expression.

Table S2. External validation on GDSC cervical cancer cell lines using PRISM trained models.

Method	MSE	RMSE	MAE	R^2	PCC	SCC
XGB	0.0105	0.1025	0.0801	0.4721	0.6214	0.6929
SVM	0.0113	0.1063	0.0830	0.4318	0.6199	0.6701
RF	0.0096	0.0980	0.0748	0.5382	0.6854	0.7039
LR	0.0188	0.1370	0.1045	0.1052	0.4299	0.5918
Lasso	0.0199	0.1410	0.1074	0.0741	0.4030	0.5643
BANDPR	0.0098	0.0990	0.0760	0.5281	0.7182	0.7664
DeepCDR	0.0095	0.0975	0.0742	0.5355	0.7310	0.7581
GADRP	0.0102	0.1010	0.0773	0.5112	0.7086	0.7498
MKDR-Teacher	0.0090	0.0949	0.0718	0.5783	0.7452	0.7765
MKDR-Student	**0.0080**	**0.0894**	**0.0692**	**0.6293**	**0.7682**	**0.7867**

To test cross-dataset generalizability, the MKDR-Student model trained on PRISM was applied to the SISO cervical cancer cell line in GDSC, and Drug response predictions for 153 compounds were evaluated.

Table S3. External validation on TCGA-CESC patient data for drug sensitivity classification

Method	Accuracy	Precision	Recall	F1-score	AUC
XGB	0.568	0.561	0.565	0.554	0.611
SVM	0.572	0.567	0.570	0.561	0.589
RF	0.586	0.578	0.582	0.574	0.602
LR	0.541	0.532	0.540	0.529	0.598
Lasso	0.534	0.520	0.533	0.512	0.581
BANDPR	0.582	0.574	0.579	0.566	0.612
DeepCDR	0.590	0.581	0.586	0.571	0.608
GADRP	0.585	0.573	0.580	0.564	0.620
MKDR-Teacher	0.604	0.596	0.598	0.587	0.633
MKDR-Student	**0.613**	**0.605**	**0.608**	**0.594**	**0.635**

We assessed the clinical relevance of MKDR using TCGA-CESC cervical cancer patients with matched RNA-seq profiles and treatment response annotations. In the cisplatin-treated subgroup, MKDR-Student effectively distinguished responders from non-responders based on predicted drug sensitivity scores.

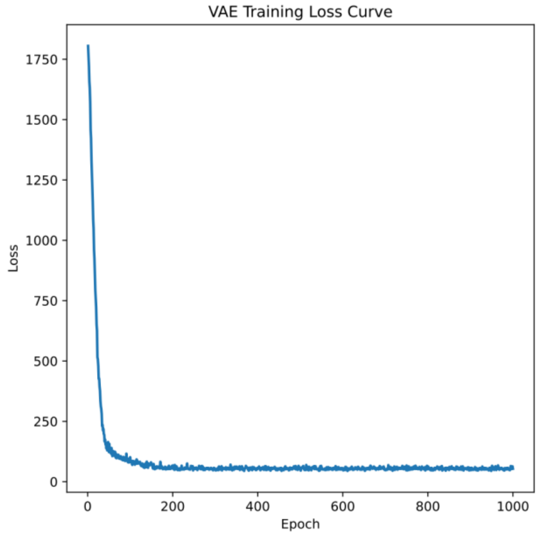

Figure S1-A. VAE training loss curve over 1000 epochs, indicating stable convergence and robust learning dynamics.

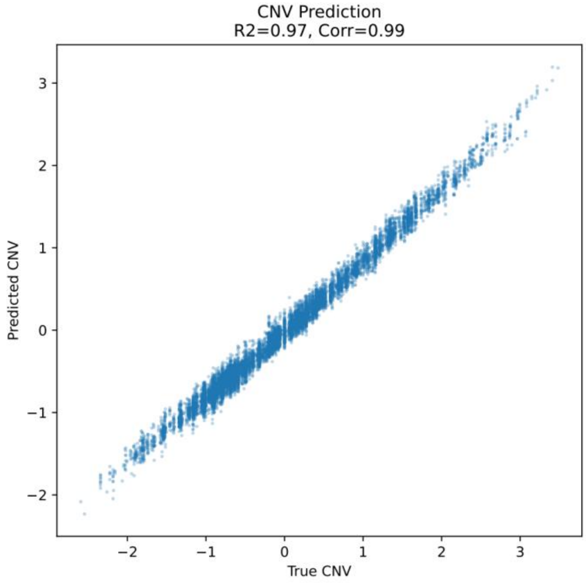

Figure S1-B. Scatter plot showing the alignment between true and reconstructed CNV values (R^2 = 0.97, PCC = 0.99), confirming strong linear reconstruction fidelity.

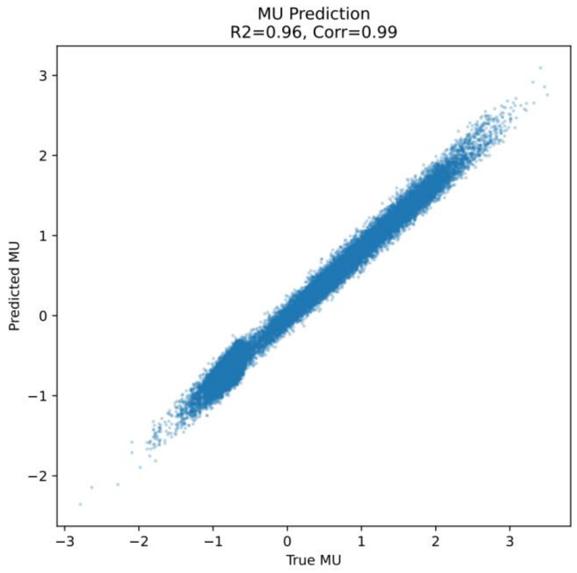

Figure S1-C. Scatter plot for mutation prediction, showing similarly strong agreement (R^2 = 0.96, PCC = 0.99), indicating that binary-like mutation features are effectively recovered from gene expression input.

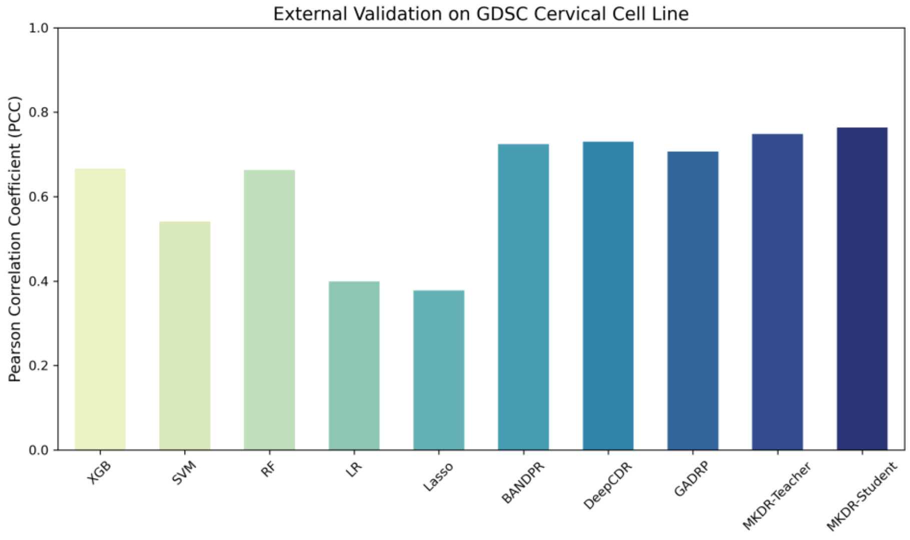

Figure S2. Pearson correlation coefficient (PCC) comparison across baseline methods on the GDSC SISO cervical cancer cell line. MKDR-Student achieves the highest PCC in this external validation.

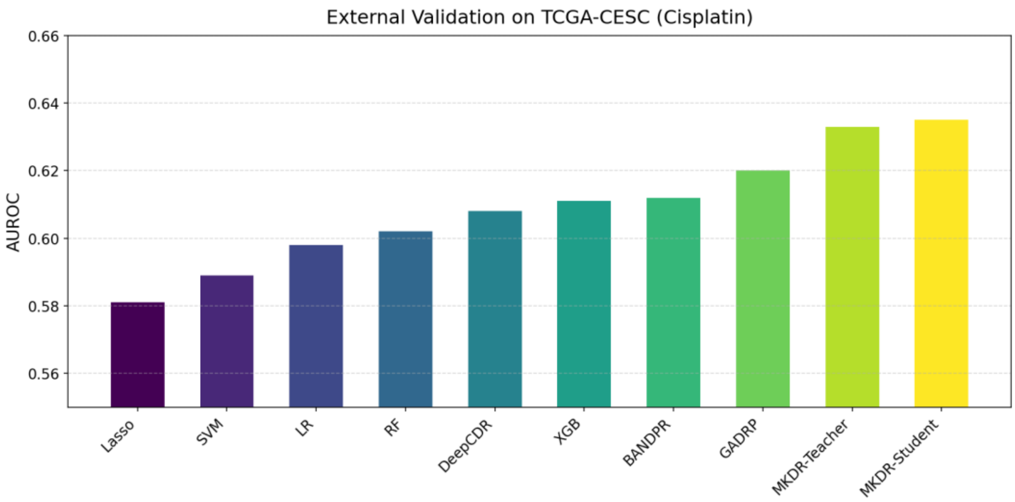

Figure S3. AUROC comparison across baseline methods and MKDR on the TCGA-CESC Cisplatin-treated cohort.
